# Vibroarthrographic Signal Spectral Features in 5-Class Knee Joint Classification

**DOI:** 10.3390/s20175015

**Published:** 2020-09-03

**Authors:** Adam Łysiak, Anna Froń, Dawid Bączkowicz, Mirosław Szmajda

**Affiliations:** 1 Faculty of Electrical Engineering, Automatic Control and Informatics, Opole University of Technology, 45-758 Opole, Poland; a.fron@doktorant.po.edu.pl (A.F.); m.szmajda@po.edu.pl (M.S.); 2 Faculty of Physical Education and Physiotherapy, Opole University of Technology, 45-758 Opole, Poland; d.baczkowicz@po.edu.pl

**Keywords:** vibroarthography, VAG, knee joint, spectral features, frequency analysis, non-invasive examination

## Abstract

Vibroarthrography (VAG) is a non-invasive and potentially widely available method supporting the joint diagnosis process. This research was conducted using VAG signals classified to five different condition classes: three stages of chondromalacia patellae, osteoarthritis, and control group (healthy knee joint). Ten new spectral features were proposed, distinguishing not only neighboring classes, but every class combination. Additionally, Frequency Range Maps were proposed as the frequency feature extraction visualization method. The results were compared to state-of-the-art frequency features using the Bhattacharyya coefficient and the set of ten different classification algorithms. All methods evaluating proposed features indicated the superiority of the new features compared to the state-of-the-art. In terms of Bhattacharyya coefficient, newly proposed features proved to be over 25% better, and the classification accuracy was on average 9% better.

## 1. Introduction

The knee is one of the most loaded joints within the human body, highly susceptible to injuries and an increased risk of early degeneration of the articular surfaces. Classical radiography is a basic diagnostic tool for imaging knee injuries. In advanced degeneration of this joint, X-ray examination correlates with arthroscopy evaluation which is used as a “gold standard”. However, the lower sensitivity and specificity of X-rays are limitations for diagnosis of early stages of chondral disorders, e.g., chondromalacia. On the other hand, the availability of modern imaging methods such as magnetic resonance imaging is limited due to high expense. One of the experimental methods developed for sensitive assessment of articular function is vibroarthrography (VAG) [1,2,3]. This relatively cheap and potentially widely available non-invasive method is based on the analysis of high frequency vibroacoustic emission, which is a natural phenomenon acquired from the relative motion of articular surfaces of the synovial joint. Although the VAG method is still under development, it reveals high accuracy, sensitivity, and specificity. Previously, it has been used to differentiate disorders of the patellofemoral joint, due to the specific, disorder-related character of the VAG signal pattern [4,5,6].

**Figure 1:**
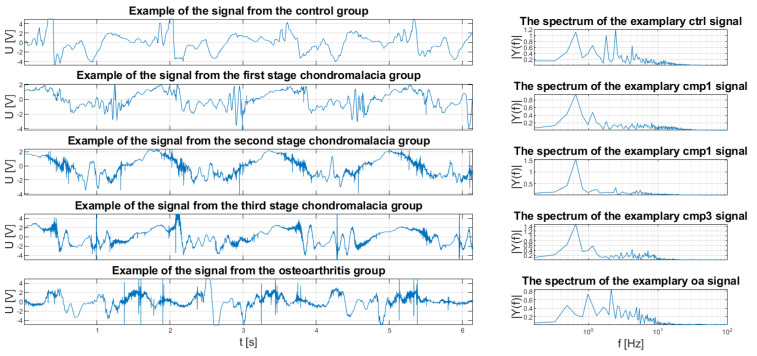
Exemplary signals from each condition group. The differences between signals results from the differences in condition of articular surfaces of the synovial joints.

Exemplary VAG signals, generated by knee joints with different conditions were presented in Figure 1. Conditions included in the Figure and in the rest of this paper are as follows: control group (healthy knee joint, abbreviated as ctrl), three stages of chondromalacia patellae (cmp1, cmp2, and cmp3, consecutively), and osteoarthritis (oa). As it can be seen, a lot of information about the specific condition could potentially be embedded in the frequency spectrum of the signal.

Although frequency analysis is present in literature devoted to vibroarthrography, the state-of-the-art spectral features are designed to distinguish all classes at once [1,2,3,4,5,6], i.e., to utilize a single value to differentiate between specific conditions. The objective of this study was to find better frequency ranges, focusing on the distinction between each particular class pairs (i.e., ctrl-cmp1, ctrl-cmp2, ctrl-cmp3, …, cmp3-oa).

This results in ten spectral features, instead of one. There are ten spectral features, as there are two classes in a pair and five distinct classes, i.e., 5 choose 2 combinations. Specific combinations were presented in Table 1, Table 2 and Table 3. That way, a single numeric value was changed to a 10-element feature vector, distinguishing conditions more precisely, ensuring that each class is described by the feature set as unambiguously as possible. That approach prevents the potential problem of some values of the feature being typical for more than one condition, making it impossible to state a precise diagnosis.

To amplify the higher frequencies in a way and enable better distinction between classes with potentially useful information hidden in wider frequency spectrum, analysis of the derivative of the signal was conducted.

## 2. Standard Descriptors

Wu in [7] specified three main methods of the knee joint VAG signal analysis: the spatiotemporal, time-frequency, and statistical analysis methods. Using the spatiotemporal analysis method Wu recommends to use adaptive segmentation [8,9] to avoid redundancy in given segments, and emphasizes its usefulness for calculating sets of features and signal classification. The statistical analysis is often used to determine distribution measures and statistical parameters [10,11], giving the possibility to display data in the form of a tabular summary [12] and graphic forms such as the histogram [6] or the box plot [13,14,15], and to analyze variations using various types of tests (t-test [16], Kruskal–Wallis test [13], one-way ANOVA test [4], Wilcoxon rank-sum test [11]). The time-frequency analysis and the frequency analysis supported by using statistical analysis are commonly used in the field of signal processing. The frequently used VAG signal analysis is the short-time Fourier transform (STFT) extended by the visual representation of a spectrogram. Łysiak et al. [14] used STFT and spectrograms, on whose specific analysis the new three descriptors have been proposed. Dołęgowski et al. [15] proposed the incremental decomposition of voltage in time and the spectrogram as the methods to identify the knee joint disease stage and compared the statistical parameters of normalized energy values of the band 50–250 Hz (P1) and 250–450 Hz (P2). Befrui et al. [2] in their measurement system used the two accelerometers, the piezoelectric disk and the potentiometer (four channels) in their measurement system. The signals were acquired at 16 kHz sampling frequency and extension and flexion cycles were extracted by using semi-automatic segmentation. The power spectra were calculated, frequency features were normalized and averaged, and the classification by a linear support vector machine (SVM) using the knee-specific feature vectors was performed.

Wavelet transformation is another tool which gives the possibility to analyze the change of signal frequency in the function of time. Mascarenhas et al. [17], for the analysis of VAG signals, proposed in their paper the tunable Q wavelet transform (TQWT) in comparison to the traditional wavelet packet decomposition (WPD). To overcome the imbalance set problem they used the synthetic minority oversampling technique (SMOTE) and afterwards they compared the performance of the random forest classifier (RF) they used to the soft margin support vector machine (SVM).

Nalband et al. [18] proposed for the VAG signals analysis a CAD system using time-frequency analysis and nonstationary signal processing techniques. Their methods were the Wavelet packet decomposition algorithm (WPD), the smoothed pseudo Wigner–Ville distribution (SPWVD) as a nonstationary time-frequency analysis, and a modified version of Hilbert–Huang transform (HHT) where instead of empirical mode decomposition (EMD) for computing intrinsic mode functions (IMF) they proposed complete ensemble empirical mode decomposition with adaptive noise (CEEMDAN). The Least square support vector machine (LS-SVM) was used.

Bączkowicz et al. [1,4] in their research presented the frequency characteristics of VAG signals by STFT. The spectra were obtained by computing Discrete Fourier Transform (DFT) of the segments (150 samples/segment), the Hanning window, and 100 samples overlap/segment. They analyzed spectral activity by summing spectral power in two different bands. The first parameter P1 concerned the range of 50–250 Hz, and the second P2 the range of 250–450 Hz. In [1] Bączkowicz et al. obtained two additional parameters by computing power of spectral density using Fast Fourier Transform (FFT): F470 at 470 Hz, and F780 at 780 Hz. They performed 2-class classification (normal/abnormal) and 5-class classification (healthy knee, the first to the third stages of chondromalacia, osteoarthritis), used the genetic search algorithm to select the best features of VAG signals, and applied four different algorithms to classify the selected features, wherein one signal feature was distinguishing all classes at once.

## 3. Enhanced Descriptors

As can be seen in Figure 1, VAG signals of different conditions consist of different frequency distortions, which could be potentially used as the features to classify them. The question arises of which frequency ranges would differentiate those classes in the best possible manner. Another question concerns the method of measuring the quality of obtained features.

### 3.1. Quality Measure of the Feature

Boxplot, as a visualization tool, gives very intuitive concept of the quality of a feature. It shows the variation of some numerical data, enabling the visual comparison. Particularly, it shows the median of a sample (indicated by the red line), interquartile range (indicated by the blue box), variability outside the interquartile range (indicated by the whiskers) and outliers (indicated by the red crosses). The question arises, though, how to quantitatively compare two features visualized on the boxplot? Lots of different measures are available in literature [19]. One of the most straightforward ones is the Bhattacharyya coefficient.

In a brief preliminary research, the Bhattacharyya coefficient was compared to several different coefficients (some existing in literature, like DBM/OVS [20,21,22,23] or Jaccard index [24], some newly defined ones). This comparison was conducted in the following way:

- The best frequency ranges were generated by every coefficient.
- Obtained frequency ranges were used to train 10 different classifiers (two decision trees, LDA, naïve Bayes, SVM, two knn classifiers, two random forests and a neural network).
- The largest mean classification accuracies were compared.
- The Bhattacharyya coefficient proved to be the best coefficient in this application.

It is defined as [25]:

(1) $$B_{coef}\left(p,q\right)=\int^{\;}\sqrt{p\left(x\right)\cdot q\left(x\right)}dx,$$

where *p* and *q* are probability distributions of two current classes and *x* is a domain of current feature values. The probability densities were obtained using kernel density estimation with a window:

(2) $$h={\left(\frac{4}{3n}\right)}^{1/5}\sigma ,$$

as defined in [26].

The Bhattacharyya coefficient indicates the overlap between two statistical samples. It ranges from 0 when distributions are completely separated to 1, when distributions overlap entirely. Therefore, in our study, the coefficient is minimized. The coefficient is equal to 0.401 for the g example from Figure 7 and 0.876 for the j.

### 3.2. Optimal Frequency Range

**Figure 2:**
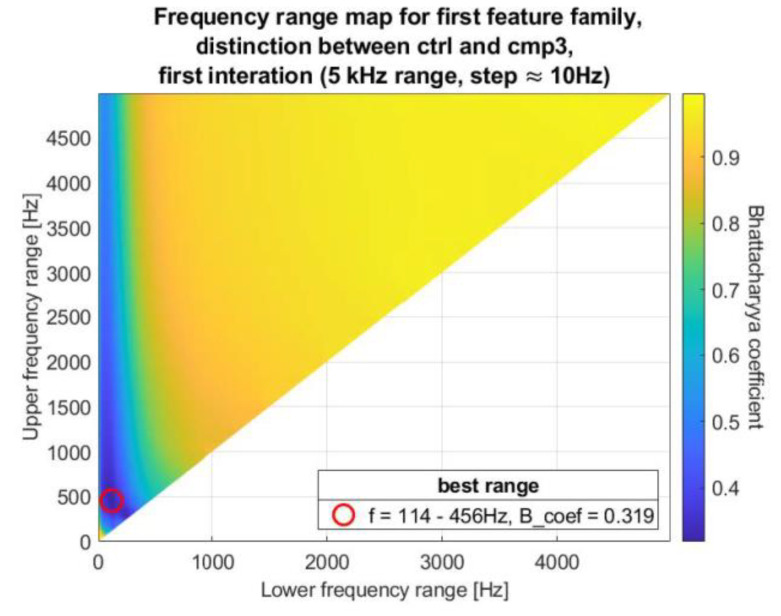
Example of the frequency range map (FRM).

Finding frequency ranges in a strict brute-force manner would be too computationally expensive, since the DFT of the analyzed signal is composed of too many samples. Consequently, the search was done in three iterations, starting sparsely in the whole frequency domain, gradually narrowing the searched range and increasing precision. To visualize the results, a kind of map was generated, called in the rest of this paper the frequency range map (FRM). An exemplary map is shown in Figure 2.

In the FRM, the abscissa x-axis is the lower frequency range and the ordinate is the upper frequency range. As a result, in the first iteration, only the upper-left half of the map consists of possible results. Applicate, or color, indicates quality of feature, defined as the Bhattacharyya coefficient. Similar maps were defined and used in [2], but with lower resolution and for slightly different features. The z-axis, or feature evaluation coefficient used in [2] was the area under the ROC curve, not the Bhattacharyya coefficient. Consequently, the coefficient in [2] was maximized, not minimized.

On each map, the best frequency range was emphasized by a red circle. The exemplary FRM in Figure 2 shows that the best range for this (first) iteration is 114–456 Hz, in which neighboring consecutive iteration will be conducted.

### 3.3. Definition of the Features

Four types or “families” of features were defined. The first family is the sum of some frequency range of the discrete Fourier transform of the VAG signal:

(3) $$d_{1}=\delta f\overset{f_{2}}{\underset{i=f_{1}}{\sum }}DℱT\left(s_{VAG}\right)\left(f_{i}\right),\;where$$

*d*_1_ is the first feature family,
*δf* is the normalization factor, equal to the $\frac{1}{f_{2}-f_{1}}$. It ensures that the value of the feature is affected only by the shape of the spectrum and not by its size,
*f*_1_ is the lower frequency range,
*f*_2_ is the upper frequency range,
*f*_2_ is the upper frequency range,
*s_VAG_* is the VAG signal,
*DFT* is the Discrete Fourier Transform operator,
*f_i_* is the *i*-th frequency amplitude.

**Figure 3:**
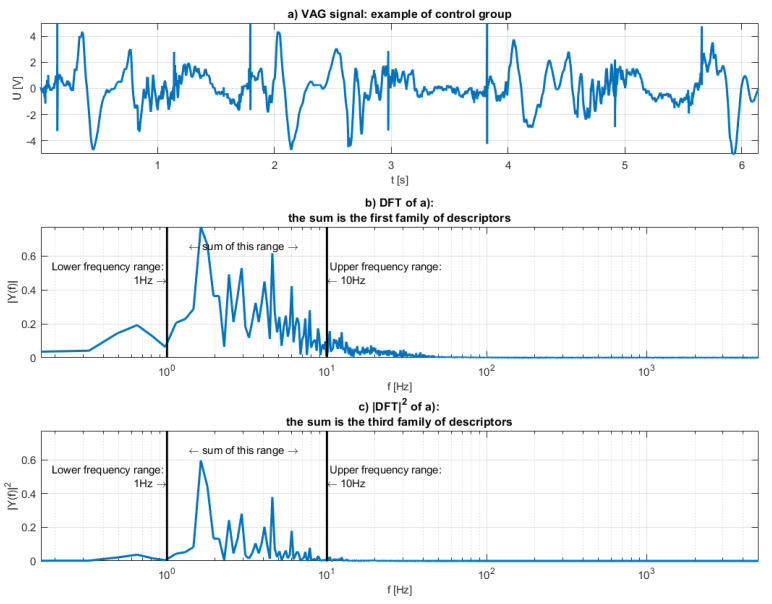
Visualization of the first and the third family of features: (**a**) the analyzed VAG signal, (**b**) the spectrum of the (**a**,**c**) the squared spectrum.

The visualization of the first feature family is provided in the (b) plot of Figure 3.

The second family of features was defined similarly, but instead of the signal, the derivative of the signal is used to obtain DFT. One of the fundamental traits of the Fourier transform is that the transform of the derivative is the transform of the original signal multiplied by the frequency:

(4) $$ℱ\left(f^{\prime }\right)\left(\xi \right)=\;2\pi i\xi \cdot ℱ\left(f\right)\left(\xi \right)$$

where ℱ is the Fourier transform. The derivative can be then interpreted as high-pass filter. The motivation for using the derivative was the certainty that potentially useful information, which could be embedded in wider frequency spectrum, will not be omitted. The second feature family was then defined as

(5) $$d_{2}=\delta f\overset{f_{2}}{\underset{i=f_{1}}{\sum }}DℱT\left\{s_{VAG}^{\prime }\right\}\left(f_{i}\right)=\delta f\overset{f_{2}}{\underset{i=f_{1}}{\sum }}\left(f_{i}\cdot 2\pi i\right)\cdot DℱT\left\{s_{VAG}\right\}\left(f_{i}\right)$$

**Figure 4:**
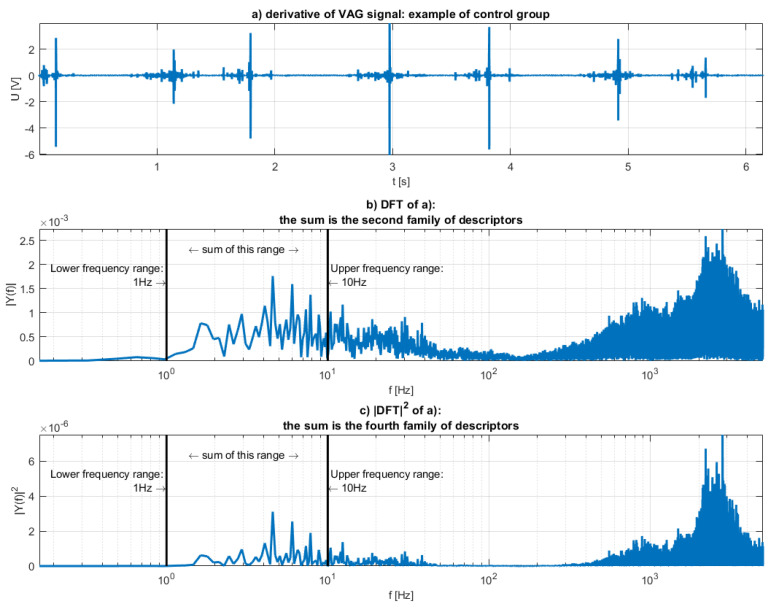
Visualization of the second and the fourth family of features: (**a**) the derivative of the analyzed VAG signal, (**b**) the spectrum of the (**a**,**c**) the squared spectrum.

The derivative and this family of features were plotted on a and b of Figure 4 respectively.

The third (Equation (6)) and the fourth (Equation (7)) families of features were defined like the first and the second, but instead of summing the DFT amplitudes, the energy was summed (so the square of the values of the previous feature families):

(6) $$d_{3}=\delta f\overset{f_{2}}{\underset{i=f_{1}}{\sum }}{\left|DℱT\left(s_{VAG}\right)\left(f_{i}\right)\right|}^{2},$$

(7) $$d_{4}=\delta f\overset{f_{2}}{\underset{i=f_{1}}{\sum }}{\left|\left(f_{i}\cdot 4\pi^{2}i\right)\cdot DℱT\left\{s_{VAG}\right\}\left(f_{i}\right)\right|}^{2}.$$

The illustrations of those families were shown in Figure 3c and Figure 4c respectively.

Interpretation of the derivative of the signal as a high pass filter can clearly be seen in Figure 3 and Figure 4. Higher frequencies are much more potent in Figure 4, while lower frequencies seem relatively unaltered. Energies, or the c plots of both Figures appear “sharper”. It is to be expected, as the energy is the square of the spectrum.

The calculation of the feature values was followed by the evaluation by Bhattacharyya coefficient. The point with coordinates: x = lower frequency range, y = upper frequency range, z = the Bhattacharyya coefficient of the feature generated by x-y frequency range, is then plotted on FRM. Then, the next feature, composed of slightly different frequency range is evaluated, plotted, etc.

## 4. Research Methodology

**Figure 5:**
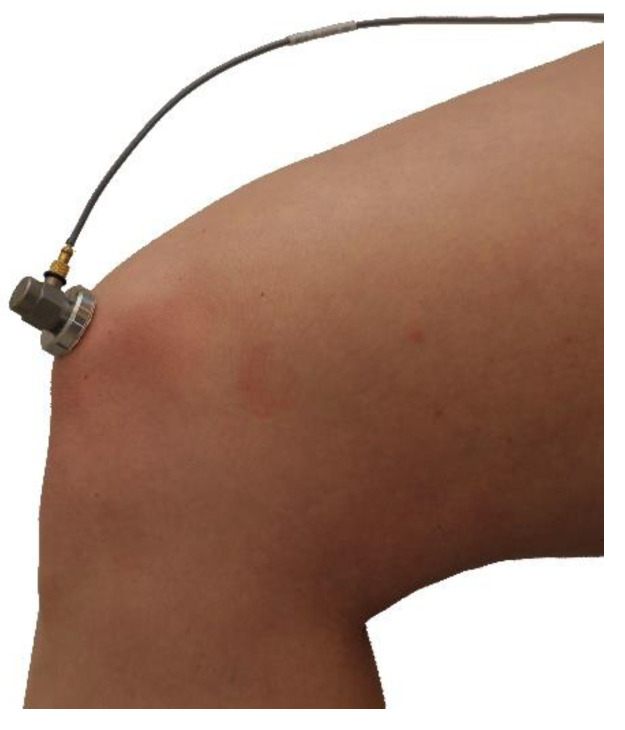
The accelerometer attachment: 1 cm above the apex of patella.

For the purposes of this research, VAG signals were acquired from groups of patients qualified by specialists as having first stage chondromalacia (cmp1), second stage chondromalacia (cmp2), third stage chondromalacia (cmp3), and osteoarthritis (oa) and from the control group, healthy knee (ctrl). All subjects underwent routine medical interviews, physical examination, and imaging via MRI. Patients with stages I–III of chondromalacia patellae were classified in accordance with the Outerbridge classification [27]. In turn osteoarthritis (OA) patients were diagnosed with mild to moderate knee OA (grade II and III according to the Kellgren-Lawrence grading system [28]) with a disease duration of more than 2 years. All imaging examinations were analyzed by single radiologist, who was blinded to patients’ symptoms. To prevent any signal artifacts from deteriorations other than chondral lesions, individuals with a history of knee fracture, knee surgery, history of meniscal tears, significant knee instability, or patellar maltracking were not enrolled in the study. Moreover, due to the methodology of the VAG assessment, individuals with a restricted knee joint range of motion (required 0° to 100°), significantly weakened muscles, and substantially swollen knees in the affected lower limb were excluded from the study. The VAG examination was performed in a sitting position and consisted of exactly four full cycles of alternating extension and flexion of the knee joint (90°–0°–90°) lasting 6 s. Mounted 1 cm above the apex of patella, the acceleration sensor was recording vibration and acoustic processes occurring during the knee movements. The acceleration sensor attachment was shown in Figure 5.

After acquiring the signals, their spectra were obtained, and four feature families were proposed. For each family, a set of features containing different frequency ranges was generated, and every feature from this set was evaluated by the Bhattacharyya coefficient. The best features constructed final 10-element vector. This vector was compared to the state-of-the-art feature vector [1], by using both of those vectors as an input to some classification algorithms. The accuracies of trained algorithms determined which feature vector is superior.

### 4.1. Acquisition of the VAG Signal

The signals analyzed in this paper were obtained from knee joints of patients diagnosed by physicians into five groups: control group (containing 66 signals), I stage chondromalacia patellae (26 signals), II stage chondromalacia patellae 30 signals), III stage chondromalacia patellae (36 signals), and osteoarthritis (26 signals). The diagnoses were based on the X-ray.

### 4.2. The Process of Defining New Frequency Features

For every sample of 184 VAG signals, the frequency analysis was conducted, resulting in four spectral vectors:

- the DFT of the VAG signal, from which the first feature family is obtained, using Equation (3),
- the DFT of the derivative of the VAG signal, from which the second feature family is obtained, using Equation (5),
- squared DFT of the VAG signal, from which the third feature family is obtained, using Equation (6),
- squared DFT of the derivative of the VAG signal, from which the fourth feature family is obtained, using Equation (7).

To generate FRMs, Bhattacharyya coefficient was calculated for the sums of desired frequency ranges. As already mentioned, evaluating all possible frequency ranges would be too computationally expensive, therefore the search was done in three iterations. The search started from the full frequency range (0–5 kHz) with quite big frequency step and gradually narrowing the range and reducing the step. The last iteration was done with highest precision (the smallest frequency step) in quite narrow frequency range. Specifically, three iterations were conducted as follows:

1. the first one in range 0–5 kHz (since signal was sampled with the frequency of 10 kHz), with about 10 Hz step. Features were then defined as sums, defined by Equations (3) and (5)–(7), in subsequent ranges: 0–10 Hz, 0–20 Hz, …, 0–5000 Hz, 10–20 Hz, 10–30 Hz, …, 4980–4990 Hz, 4980–5000 Hz, 4990–5000 Hz. Every range was evaluated by Bhattacharyya coefficient and plotted as a point on the FRM.
2. the second iteration was conducted in range ±800 Hz from the best range obtained in previous iteration with about 2.5 Hz step,
3. the last iteration was conducted in range ±80 Hz from the best range obtained in previous iteration with about 0.16 Hz step.

**Figure 6:**
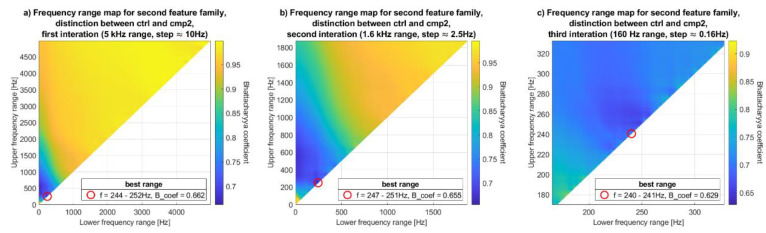
Three examples of the FRM; all of them were generated for the second family of features (i.e., the sum of the DFT of the derivative of the VAG signal). (**a**–**c**) are the consecutive iterations.

Three exemplary maps were shown on Figure 6, illustrating three consecutive iterations.

The FRMs were separately generated for every class combination (details in Table 1, Table 2 and Table 3), for every feature family, so 120 maps in total, 40 maps for final iteration. Generating separate maps for each class combination may seem redundant. Only distinction between neighboring classes appears to be sufficient. Different approach, i.e., temporary ensemble all classes but one, generating maps only for distinguishing between, for example, the second stage chondromalacia condition and the rest, generating characteristic features for each class also seems enough. As it certainly would be less computationally expensive, it probably would be less robust. Neighboring classes are not completely separate and independent, as the first stage chondromalacia precedes the second stage etc. Consequently, it would be quite difficult to find the frequency range typical for particular class only. Minimizing Bhattacharyya coefficient for every class pair ensures two things:

- that the found frequency ranges for each classes are as optimal as possible, without sacrificing the distinction between more different conditions (maybe one frequency range would be appropriate for the distinction between the first and the second stage chondromalacia, but not as effective with distinguishing between first stage chondromalacia and the osteoarthritis; the question would arise, which distinction should be dominant, and which should be sacrificed),
- that the obtained frequency ranges provide as unambiguous distinction as possible; the situation can be imagined in which two classes neighboring one another (for example cmp1 and cmp3 neighboring cmp2) can be distinguished from the one with very similar frequency ranges. Then the statement that the particular frequency range is typical for, e.g., cmp2 would be true, but the contrary would not unambiguously point out cmp1 or cmp3.

Analysis of the final 40 maps led to definition of 10 frequency ranges in different feature families, indicated by bolded font in the Table 2. Obtained features were compared to standard frequency descriptors defined in [1].

### 4.3. The Verification of Defined Features as a Classifier Input

The comparison between newly defined features and the state-of-the-art was done in two ways. First, the Bhattacharyya coefficients of features were compared. However, as mentioned before, a 4-element (P1, P2, F470, F780) spectral feature vector was proposed in [1]. In this study, 10 features were proposed, making it hard to directly compare the quality of obtained features.

To compare obtained features, both newly defined and state-of-the-art were used to train a couple of classifier algorithms. Utilized algorithms were listed in Table 4. To find which feature vector is superior, the final classification accuracies were compared.

The classification accuracy of every classifier strongly depends on the division of the data into the teaching, testing, and validation sets. To surpass that potential bias, 1024 classifiers of each type were constructed with random division of the data into mentioned sets.

## 5. Results and Discussion

The results obtained for every combination of classes, for each feature family, were presented in Table 1 and Table 2. The use of the derivative was quite fruitful for some class pairs, even though intuition behind it, i.e., the informativeness of higher frequency spectrum, was not fully correct. For example, the distinctions between the first and the second stage chondromalacia or the third stage chondromalacia and osteoarthritis were better with the use of the derivative, but the range of the frequencies was very narrow. Generally, the most optimal results obtained for the primitive VAG signal and its derivative are comparable.

**Table 1:** Research results: the Bhattacharyya coefficients, for best frequency range, for each feature family, for each class combination. Corresponding frequency ranges are presented in Table 2.

Boxplot Letter (Figure 7)	Class Combination	The Bhattacharyya Coefficient for Feature Family:			
		1 (DFT of the Signal)	2 (DFT of the Derivative)	3 (Square of the DFT of the Signal)	4 (Square of the DFT of the Derivative)
a	ctrl, cmp1	0.863	0.844	0.842	0.872
b	ctrl, cmp2	0.634	0.629	0.659	0.662
c	ctrl, cmp3	0.318	0.316	0.453	0.464
d	ctrl, oa	0.308	0.367	0.458	0.462
e	cmp1, cmp2	0.726	0.717	0.769	0.768
f	cmp1, cmp3	0.446	0.442	0.560	0.577
g	cmp1, oa	0.401	0.486	0.587	0.603
h	cmp2, cmp3	0.637	0.667	0.694	0.693
i	cmp2, oa	0.594	0.696	0.741	0.744
j	cmp3, oa	0.900	0.919	0.897	0.876

**Table 2:** Research results: the best frequency range for each feature family, for each class combination. Corresponding Bhattacharyya coefficient values are presented in Table 1.

Boxplot Letter (Figure 7)	Class Combination	The Frequency Range (Hz) for Feature Family:			
		1 (DFT of the Signal)	2 (DFT of the Derivative)	3 (Square of the DFT of the Signal)	4 (Square of the DFT of the Derivative)
a	ctrl, cmp1	235.51–235.51	279.95–279.95	235.51–235.51	226.56–226.56
b	ctrl, cmp2	240.23–240.56	240.23–240.56	331.22–331.38	331.22–331.38
c	ctrl, cmp3	111.17–452.96	103.19–359.21	109.70–428.39	26.20–303.71
d	ctrl, oa	15.79–1110.68	0.00–554.85	47.69–5000.00	0.00–649.25
e	cmp1, cmp2	398.11–398.93	405.76–405.76	258.3–258.63	239.10–239.10
f	cmp1, cmp3	78.78–465.82	15.14–417.97	78.61–428.39	9.11–290.36
g	cmp1, oa	8.46–849.61	0.81–470.70	42.64–5000.00	0.81–690.92
h	cmp2, cmp3	94.40–394.86	91.80–287.43	88.05–392.42	79.92–263.83
i	cmp2, oa	8.79–955.73	0.00–513.02	71.45–5000.00	233.40–233.89
j	cmp3, oa	0.00–4384.44	0.98–166.18	16.28–193.20	157.88–157.88

**Figure 7:**
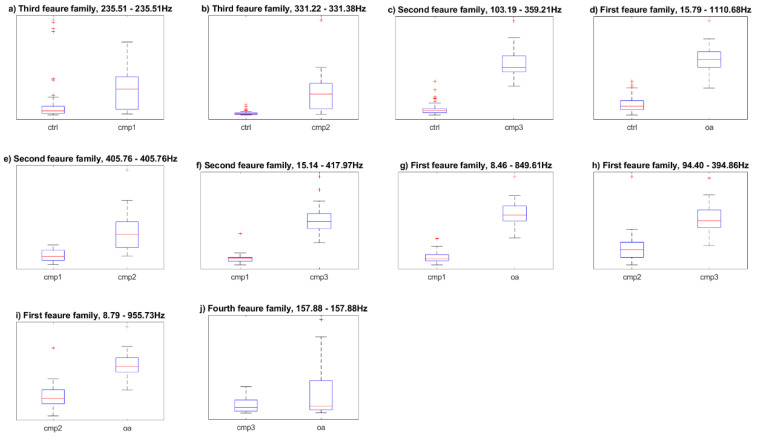
Boxplots of the best results for each class combination. The class distinctions are following: (**a**) ctrl-cmp1, (**b**) ctrl-cmp2, (**c**) ctrl-cmp3, (**d**) ctrl-oa, (**e**) cmp1-cmp2, (**f**) cmp1-cmp3, (**g**) cmp1-oa, (**h**) cmp2-cmp3, (**i**) cmp2-oa, (**j**) cmp3-oa. All of the letters correspond with Table 1 and Table 2.

Boxplots of the best results for each combination of classes were shown in Figure 7.

The new features were defined with the emphasis on distinction between each particular class pair. Then, it can be seen, that some of the pairs are well distinguished, especially when the knee joint conditions strongly differ (for example, the control group and the third stage chondromalacia or the first stage chondromalacia and the osteoarthritis). The problem persists, though, with the distinction between neighboring pairs, especially the third stage chondromalacia and the osteoarthritis. This is to be expected to some extent, as the chondromalacia condition commonly occurs with the osteoarthritis.

To better evaluate the quality of the obtained features, they were compared to the features defined in [1] (used signal database was the same in this research and in [1]). Firstly, the Bhattacharyya coefficient was generated for the frequency features from [1], then some classification algorithms were used to quickly check potential classification accuracies, using both the new features and the features defined in [1].

**Figure 8:**
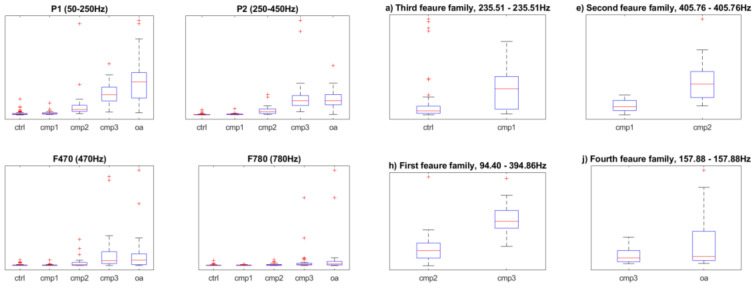
Boxplots of the features defined in [1] compared to boxplots of the new features defined for neighboring classes. The letters in titles of the new features correspond with Table 1 and Table 2.

The comparison between features defined in [1] and features defined in this paper was presented in Table 3. The boxplots of the features from [1] were given in Figure 8.

**Table 3:** The Bhattacharyya coefficients of frequency features defined in [1] compared to present study. The improvement is calculated as $\frac{\left(B_{{coef}_{OLD}}-B_{{coef}_{NEW}}\right)}{B_{{coef}_{OLD}}}\cdot 100$.

Class Combination	Bhattacharyya Coefficient					
	P1 (50–250 Hz)	P2 (250–450 Hz)	F470 (470 Hz)	F780 (780 Hz)	The Best Feature from Table 1	Improvement (%)
ctrl, cmp1	0.964	0.960	0.951	0.963	0.842	11.46
ctrl, cmp2	0.906	0.795	0.924	0.943	0.659	17.11
ctrl, cmp3	0.549	0.668	0.837	0.947	0.316	42.44
ctrl, oa	0.582	0.516	0.860	0.943	0.308	40.31
cmp1, cmp2	0.942	0.874	0.936	0.904	0.717	17.96
cmp1, cmp3	0.607	0.782	0.882	0.955	0.442	43.48
cmp1, oa	0.670	0.635	0.901	0.948	0.401	36.85
cmp2, cmp3	0.809	0.881	0.952	0.965	0.637	21.26
cmp2, oa	0.809	0.809	0.950	0.951	0.594	26.58
cmp3, oa	0.924	0.962	0.985	0.980	0.876	5.19

None of the newly obtained frequency ranges was close to the range of P1 or P2 from [1]. The frequencies around 250 Hz, the interface of P1 and P2, occur in the Table 2 relatively often, however the ranges are significantly narrower. The new feature closest to F470 from [1] is feature e from Table 2, so the sum of the DFT of the derivative of the signal for the frequencies around 400 Hz. Nonetheless, the difference in frequencies is quite big. The F780 feature from [1] does not overlap with any features obtained in conducted research.

The new features are superior, in terms of Bhattacharyya coefficient, for every class combination. Improvement of distinction is quite significant for some pairs (like the pair cmp1–cmp3, for which the coefficient dropped by more than 40%), and for others is smaller (line the cmp3–oa pair, for which the coefficient dropped by around 5%).

To test the features as a diagnostic tool, ten different types of classifiers were constructed for the newly defined features and another ten classifiers for the features defined in [1]. The comparison between their accuracies is presented in Table 4.

**Table 4:** Comparison between classification accuracies for the new features and the features defined in [1]. The improvement is calculated as $\frac{\left(classification\_accuracy_{OLD}-classification\_accuracy_{NEW}\right)}{classification\_accuracy_{OLD}}\cdot 100$

No.	Classifier	Classification Accuracy			Additional info. about Classifier
		The New Features	The Features Defined in [1]	Improvement (%)	
1	decision tree	0.62	0.59	5.1	max. 10 splits
2	decision tree	0.60	0.59	1.7	max. 5 splits
3	discriminant analysis	0.63	0.53	18.9	
4	naïve Bayes	0.64	0.48	33.3	
5	support vector machine	0.67	0.63	6.3	linear kernel
6	k nearest neighbors	0.64	0.61	4.9	*k* = 20, euclidean distance
7	k nearest neighbors	0.63	0.62	1.6	*k* = 5, euclidean distance
8	decision forest	0.62	0.57	8.8	bagging
9	decision forest	0.63	0.60	5.0	boosting, max 10 splits
10	neural network	0.63	0.60	5.0	10 hidden neurons, tansig function
mean		0.63	0.58	9.1	
max		0.67	0.63	33.3	

All accuracies presented in the Table 4 are actually the mean values of 1024 classifiers of a given type.

**Figure 9:**
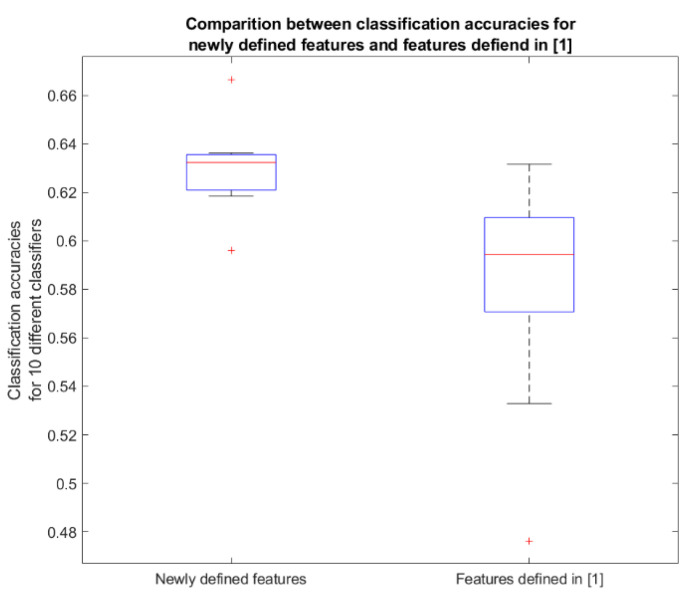
Visual representation of the data given in Table 4.

As expected, classifiers constructed on new feature set proved to be more accurate. Additional visual comparison was given with the use of boxplot on the Figure 9. All the data came from the Table 4.

Although the new features are more useful for classifying different knee joint conditions, obtaining them is more computationally expensive. Additionally, they are probably somewhat correlated to each other, which could be undesirable while using them as an input for some classification algorithms [29]. A principal component analysis could be a useful step before constructing classifier, but it would increase computational cost even further.

## 6. Conclusions

Conducted research resulted in the definition of ten new spectral features, emphasizing differences between every possible knee condition class pair. This ensured that the found frequency ranges were as optimal as possible, without the risk of sacrificing potentially valuable information about the differences between some class pairs. Proposed features were used to train ten different classification algorithms proving their superiority as signal descriptors. In comparison to [1], in terms of Bhattacharyya coefficient, newly proposed features proved to be over 25% better. The classification accuracy has increased on average by 9%.

Frequency Range Maps were proposed as a spectral feature visualization tool, utilizing Bhattacharyya coefficient as a feature quality measure. Visual analysis of those maps may help better understand the nature of the signal. The maps are not limited to VAG signals and can be used in different fields of research.

Proposed features may constitute the reference point in future studies, utilizing proposed frequency ranges as a method of measuring effect of some factor on quality of knee joint itself.
